# Author’s Response

**DOI:** 10.4269/ajtmh.19-0546b

**Published:** 2019-10

**Authors:** David Johnston

**Affiliations:** Hawaii Department of Health; Honolulu, Hawaii; E-mail: david.johnston@doh.hawaii.gov

Dear Sir,

We would like to thank Dr. Prociv for the interest in angiostrongyliasis he has shown in his letter.^1^ However, with regard to the comment that our review should have included a more in-depth analysis of the clinical aspects of angiostrongyliasis, the focus of our study was on the epidemiological aspects of the disease occurring in Hawaii and the study was not intended to be a clinical review. We agree more clinical research is needed to improve our understanding of the clinical course and management of angiostrongyliasis. A more complete understanding of both the epidemiology and the clinical aspects of the disease would help prevention and control efforts.
